# Tumor-B-cell interactions promote isotype switching to an immunosuppressive IgG4 antibody response through upregulation of IL-10 in triple negative breast cancers

**DOI:** 10.1186/s12967-022-03319-5

**Published:** 2022-03-07

**Authors:** Nicole J. Toney, Lynn M. Opdenaker, Kader Cicek, Lisa Frerichs, Christopher Ryan Kennington, Samuel Oberly, Holly Archinal, Rajasekharan Somasundaram, Jennifer Sims-Mourtada

**Affiliations:** 1 Cawley Center for Translational Cancer Research, Helen F Graham Cancer Center and Research Institute, Christiana Care Health Services, Inc., 4701 Ogletown Stanton Rd Suite 4300, Newark, DE 19713 USA; 2 Department of Biological Sciences, The University of Delaware, Newark, DE USA; 3 The Wistar Institute, Philadelphia, PA USA

**Keywords:** Breast cancer, Triple negative breast cancer, IgG4, B cells, Interleukin 10

## Abstract

**Background:**

Triple negative breast cancer (TNBC) is an aggressive breast cancer for which there is currently no targeted therapy. Tumor-infiltrating B-cells (TIB) have been observed in tumor tissues of TNBC patients, but their functional role is unclear. IgG4 is one of four antibody subclasses of IgG expressed and secreted by B cells. Unlike other IgG isotypes, IgG4 has an immunosuppressive function and is induced by Th2-type cytokines. In cancers such as melanoma, IgG4 has been linked with advanced disease and poor patient survival. Therefore, we sought to determine if IgG4 + B cells are present and determine the mechanisms driving isotype switching in TNBC.

**Methods:**

We performed co-culture assays to examine expression of Th2 cytokines by TNBC cells with and without the presence of B cells. We also performed in vitro class switching experiments with peripheral B cells with and without co-culture with TNBC cells in the presence or absence of an IL-10 blocking antibody. We examined expression of CD20^+^ TIB, IgG4 and Th2 cytokines by immunohistochemistry in 152 TNBC samples. Statistical analysis was done using Log-Rank and Cox-proportional hazards tests.

**Results:**

Our findings indicate that B cells interact with TNBC to drive chronic inflammatory responses through increased expression of inflammatory cytokines including the TH2 cytokines IL-4 and IL-10. In vitro class switching studies show that interactions between TNBC cell lines and B cells drive isotype switching to the IgG4 isotype in an IL-10 dependent manner. In patient tissues, expression of IgG4 correlates with CD20 and tumor expression of IL-10. Both IgG4 and tumor IL-10 are associated to shorter recurrence free survival (RFS) and overall survival (OS) in TNBC. In a multi-variant analysis, IL-10 was associated with poor outcomes indicating that tumor IL-10 may drive immune escape.

**Conclusions:**

These findings indicate that interactions between TIB and TNBC results in activation of chronic inflammatory signals such as IL-10 and IL-4 that drive class switching to an IgG4 + subtype which may suppress antibody driven immune responses. The presence of IgG4 + B cells may serve as a biomarker for poor prognosis.

**Supplementary Information:**

The online version contains supplementary material available at 10.1186/s12967-022-03319-5.

## Background

Triple negative breast cancer (TNBC) accounts for approximately 15% of all breast cancers and is defined by lack of expression of estrogen and progesterone receptors, and lack of overexpression of human epidermal growth factor receptor 2 (HER2). TNBC is an aggressive breast cancer subtype characteristic of having quick progression and poor patient outcomes. Patients have higher rates of early recurrence than other breast cancer subtypes, particularly in the first 5 years after diagnosis [1]. Due to the aggressive nature of TNBC, patients tend to have poor prognosis, including poor overall survival (OS), breast-cancer specific survival (BCSS), and recurrence-free survival (RFS) [1–3]. It is unclear what drives the aggressive nature of TNBC.

While genetics may play a role, the tumor microenvironment has a substantial influence on many tumor attributes. An inflammatory tumor microenvironment is often present in TNBC. Compared to other breast cancer subtypes, TNBC has a higher level of tumor infiltrating lymphocytes, including CD8 and CD4 + T cells, and CD20 + B cells [4–6]. Likewise, expression of inflammatory cytokines, immunosuppressive genes and markers of chronic inflammation are higher in TNBC than other breast cancer subtypes [4–8]. Although high densities of infiltrating lymphocytes are associated to increased response to chemotherapy and better outcomes in highly proliferative tumors, this prognostic association may be lost in tumors with lower proliferative capacity and in certain subtypes of TNBC, such as the claudin low subtype [6, 9]. Additionally, gene signatures related to chronic inflammatory responses have been associated to ER negativity, increased metastasis and poor prognosis in TNBC [4, 8, 10, 11]. Moreover, increased activation of NfKB, STAT3 and AP-1 pathways has been observed in triple negative cell lines and tumors compared to other breast cancer subtypes [11].

Chronic inflammation associated with the humoral immune response has been found to promote aggressiveness in a number of solid tumor types. B lymphocytes have been shown to drive chronic inflammation in a murine model of inflammation-associated epithelial cancer, contributing to tumor-promoting processes such as an angiogenesis, epithelial cell proliferation, and further recruitment of immune cells [12]. B lymphocyte-derived factors have also been shown to upregulate proteins and transcription factors involved in pro-inflammatory signaling pathways such as IKK complex proteins and STAT3 in tumor cells, resulting in tumor-promoting processes such as inflammation and angiogenesis [13–18]. Moreover, TIB cells were found to induce chronic inflammation in melanoma, leading to activation of the inflammatory NfKB signaling pathway [19].

Chronic inflammation is known to induce immunosuppressive responses in both malignant and non-malignant tissues through a variety of process affecting both innate and adaptive immunity. Immune modulation of antibody responses occurs in part through class switching to the immunosuppressive IgG4 antibody subtype. This switch is driven by chronic antigen exposure and T-helper type 2 cytokines such as IL-4and IL-10 [10, 20–22] and serves to ramp down the immune system during inflammatory responses [23, 24]. Due to the unique structure of its hinge region, IgG4 has reduced effector functions compared to other IgG isotypes. These structural changes result in poor ability to bind complement and Fc receptors and to activate effector cells [23]. Furthermore, IgG4 can interact with other antibodies of the IgG (particularly IgG1) and IgE classes through their Fc domains, serving in an immunoregulatory role to decrease antibody responses [25–27].

The presence of IgG4 antibodies has been reported in a subset of cancer types, namely melanoma [28–30], extrahepatic cholangiocarcinoma [31], glioblastoma [32], pancreatic cancer [33], and hepatocellular carcinoma [34] and has been shown to have a negative correlation with recurrence free and overall survival [29, 30, 34]. Although the role of IgG4 in breast cancer is unclear, a recent study reported an enrichment of IgG + clonally expanded B cells in TNBC, with an increase in IgG4 class switching in TNBC compared to non-TNBC [35]. In this study we sought to understand the mechanisms driving IgG4 class switching in TNBC and determine the relationship of IgG4 + B cells in the TME to patient outcomes.

## Methods

### Cell culture and reagents

MDA-MB-231 cells obtained from American Type Culture Collection were maintained in RPMI-1640 medium (ThermoFisher Scientific, #11875) + 10% FBS (ThermoFisher Scientific, #10437028) and 1% antibiotic/antimycotic (ThermoFisher Scientific, #15240). SUM159 cells were obtained from Asterand (Detroit, MI) and cultured in Ham’s F12 medium (ThermoFisher Scientific, #11765) + 10% FBS, 1 × antibiotic/antimycotic, 2 μg/ml insulin (ThermoFisher Scientific, #12585) and 100 ng/ml hydrocortisone (Sigma-Aldrich, #H0135). Primary human peripheral B lymphocytes and EBV transformed B cells were grown in RPMI-1640 + CellXVivo Human B Cell Expansion Kit as described by manufacturer (R&D Systems, #CDK005), L-glutamine (ThermoFisher Scientific, #25030), 2-mercaptoethanol (ThermoFisher Scientific, #21985), Insulin, Penicillin–Streptomycin (ThermoFisher Scientific, #15070063), and 10% FBS. EBV-transformed human peripheral B cells were grown in RPMI + 10% FBS and 1 × antibiotic/antimycotic and treated with CellXVivo Human B cell expansion kit for experimental analysis.

### Isolation of PBMCs and flow cytometry

Peripheral human blood was obtained with consent from women with TNBC prior to surgical resection or treatment and peripheral blood mononuclear cells (PBMCs) were isolated using Ficoll-Paque^®^ (GE Healthcare, #95021). B lymphocytes were isolated using B Cell Isolation Kit II, human (Miltenyi Biotech, #130-091-151) according to the manufacturer’s instructions. For co-cultures, B cells and tumor cells were separated with a 0.4 μm 6-well cell culture insert with B cells in the upper chamber. Cells were cultured for 6 days, with media changed on day 3. Following co-culture, B cells were prepared for flow cytometry analysis by staining with an IgG4 antibody for 30 min in the dark (#9200-09, mouse anti-human IgG4 PE-conjugated, 1:50, SouthernBiotech). Cells were washed, resuspended in 300 μl PBS + 10% FBS, and filtered through a 35-um filter cap in a 5 ml FACS tubes to create a single cell suspension. Samples were run on the BD FACSAria II (BD Biosciences) at the Christiana Care’s Helen F. Graham Cancer Center and Research Institute core facility. For IL-10 blocking studies, anti-human IL-10 (clone JES3-97D, Biolegend) was added at a concentration of 1ug/ml, daily during co-culture. Flow cytometry experiments were repeated at least three times with each tumor cell line.

### Real-time PCR

Tumor cell lines were cultured with and without B cells for 24 h as described above and RNA was isolated. Real-time PCR was performed as previously described [36]. Samples were run on an Applied Biosystems’ 7500 Fast Real-Time PCR System with Power SYBR Green PCR Master Mix (Life Technologies), using the primers described in Table 1. All experiments were performed in at least four experimental replicates in each cell line.

**Table 1:** Primers used for qPCR

Primer	Forward Sequence (5′–3′)	Reverse Sequence (5′–3′)
VEGF	TGCAGATTATGCGGATCAAACC	TGCATTCACATTTGTTGTGCTGTAG
PDGFA	GGTGGTCACAGGTGCTTTTT	AAACCACTTAAGGCTCTCAGGA
PDGFB	TGAGAAAGATCGAGATTGTGCG	GGGCTTCGGGTCACAGG
IGF-1	CATGTCCTCCTCGCATCTCT	AGCAGCACTCATCCACGATA
IL-1beta	CTGAAAGCTCTCCACCTCCA	CCAAGGCCACAGGTATTTTG
IL-4	CACAAGCAGCTGATCCGATTC	TCTGGTTGGCTTCCTTCACAG
Il-6	GACAAAGCCAGAGTCCTTCAGAGA	CTAGGTTTGCCGAGTAGATCT
IL-8	AAGCTGGCCGTGGCTCTCTT	TGG TGG CGC AGT GTG GTC CA
IL-10	GGTTCGCAAGCCTTGTCTGA	TCCCCCAGGGAGTTCACAT
GAPDH	CCAGGTGGTCTCCTCTGACTT	GTGGTCGTTGAGGGCAATG

### ELISA

For the IL-10 ELISA, tumor cells were co-cultured with B cells for 24-h. Control cells were incubated with media only. To ensure detection of tumor cell IL-10, B lymphocytes and media were removed, and fresh media was added back to wells for a 24-h period. Supernatants were then collected, spun down to remove cell debris, and analyzed using the Quantikine IL-10 Elisa kit (R&D systems) according to manufacturer’s instructions. For the IgG4 Elisa, EBV B cells were activated and co-cultured with or without TNBC cell lines for 5 days as previously described. B cells were then removed and allowed to expand for two weeks. Cells were then plated for 24 h and supernatant was captured. Supernatants were analyzed using the Human IgG4 Elisa kit (Invitrogen). Absorbance was read at 450 nm on the Infinite^®^ 200 PRO NanoQuant (Tecan Life Sciences). Experiments were performed in duplicate in each cell line.

### Patient samples

Breast cancer tissue specimens were obtained from the Helen F. Graham Cancer Center and Research Institute (HFGCCRI) biorepository under a protocol approved by the institutional review board. Tissue was obtained from surgical resection from women who were pathologically diagnosed with TNBC and consented to the use of their tissues for research. A pathological diagnosis of TNBC is defined as less than 1% of tumor cell expression of the hormone receptors estrogen and progesterone and a negative HER finding by IHC (0 or 1 +) or negative FISH. Tissue blocks were prepared by formalin-fixation and paraffin-embedding. Tissues were constructed into 4 × 5 tissue microarrays (TMA) with a 5 mm core size and cut as serial sections. All patients underwent adjuvant radiation and chemotherapy after surgical resection.

### Immunohistochemical procedure

Serial paraffin-embedded slides were deparaffinized and rehydrated, and heat antigen epitope retrieval was performed for 16 h at 60 °C. Slides were stained using the DAB Substrate kit (Abcam, ab64238) for IL-10 and CD20 and the Mouse and Rabbit Specific HRP/AEC (ABC) Detection IHC Kit (Abcam, ab93705) for IL-4 and IgG4, following the manufacturer’s instructions. Primary antibodies were incubated overnight at 4 °C with a rabbit monoclonal antibody to IgG4 (Abcam, ab109493, 1:1000), a rabbit polyclonal antibody to IL-4 (Abcam, ab9622, 1:100), and mouse monoclonal antibodies to CD20 (Abcam, ab9475, 1:100) and IL-10 (Santa Cruz, sc-8438, 1:50). Slides were counterstained with Harris hematoxylin for 10 min. Images were captured using a Zeiss Axio microscope using a 10X objective. CD20 was scored by overlaying a 20 × 20 grid of 1 mm squares and calculating the percent of tissue-containing squares which contained 10 + lymphocytes. IgG4 was scored by counting the total number of IgG4 + B cells. For IL-10 and IL-4 staining, segmentation of tumor cells and measurement of integrated density was performed using Zen Blue. The median intensity or percentage was used to determine low vs high samples. For CD20, low, intermediate and high density was determined using the mean ± one standard deviation. Control staining was performed with an isotype matched antibody (Santa Cruz, sc-2025, sc-2027) using the same staining conditions. No staining was observed. (Additional file 1: Fig. S1).

### Statistical analysis

Statistical analysis was done in graph pad PRISM 8 and IBM27 SPSS. Relationships among scored slides was done using Spearman’s correlation analysis. Flow cytometry and real time quantitative PCR data was analyzed using student’s T-test. Survival and recurrence were analyzed using Kaplan–Meier curves and Mantel Log-Rank tests. Multi-variate analysis was carried out using Cox-proportional hazards tests.

## Results

### B cells induce chronic inflammation in TNBC

**Fig. 1:**
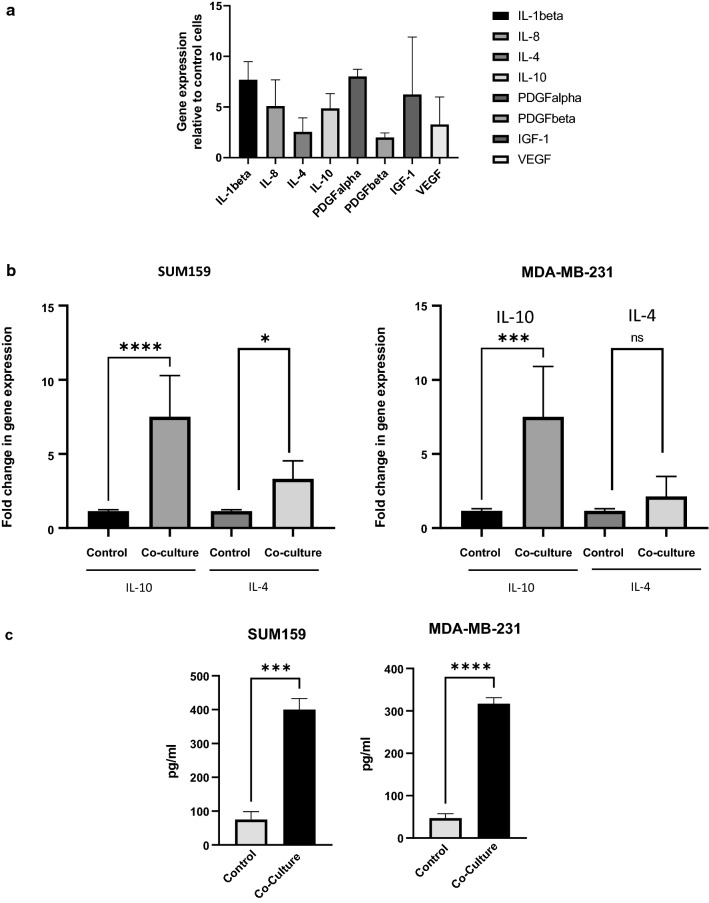
Interactions between TNBC and tumor cells result in upregulation of inflammatory cytokines in tumor cells. **a** Real-time PCR analysis of SUM159 tumor cells co-cultured with B cells showing increases in inflammatory cytokines over non-co-cultured cells. **b** SUM159 tumor cells or MDA-MB-231 cells after 24 h co-culture with B cells result in an increased gene expression of IL-10 and IL-4. Graph shows fold change of co-cultured cells compared to controls. Error bars represent standard deviations. These experiments were completed in at least triplicate. **c** Elisa showing increased protein expression (pg/ml) of IL-10 in SUM159 and MDA-MB-231 cells after 24 h co-culture with B cells. This experiment was performed in duplicate in each cell line. Error bars represent standard deviation of technical replicants. Significance is indicated by asterisk, ****p < 0.0001, ***p < 0.001, **p < 0.01, *p < 0.01, ns p > 0.05

Chronic inflammation is a hallmark of TNBC [11]. TIB cells have been associated with chronic inflammatory signatures [15–18, 37] and have been shown to secrete several inflammatory cytokines in breast cancer [38]. While previous studies have shown that tumor cells are capable of activating inflammatory signaling cascades in B cells [35, 38], the effects of this interaction on tumor cells are less clear. We therefore sought to determine the effects of B cells on tumor cells in co-culture studies with primary peripheral B cells isolated from the blood of TNBC patients. We observed increases in gene expression of several inflammatory cytokines in TNBC cells co-cultured with B cells (Fig. 1a). Of particular interest was upregulation of the TH2 type cytokines IL-10 and IL-4 in triple negative cells, which have been shown to drive IgG4 expression and are associated with immunosuppressive responses [22, 39]. To confirm these findings, we conducted a 24-h co-culture of primary human peripheral B cells isolated from women with TNBC and the TNBC cell lines MDA-MB-231 and SUM159. Real-time PCR was used to analyze changes in gene expression of IL-10 and IL-4. We found significant increases in IL-10 gene expression in both cell lines (Fig. 1b). An average 7.5-fold increase was observed in SUM159 and a 7.4-fold increase was observed in MDA-MB-231 cells co-cultured with B cells compared to controls. Increases in gene expression of IL-4 was also observed in both lines, 3.4-fold increase for SUM159 and 2.1-fold increase for MDA-MB-231, but this increase was only significant in the SUM159 cell line. To determine if this gene expression correlated to an increase in protein expression, we performed an ELISA specific for human IL-10. To determine tumor-specific production of IL-10, TNBC cell lines were co-cultured for 24 h with B cells, followed by a 24 h culture without B cells, after which media was removed and analyzed for the presence of IL-10 protein. As shown in Fig. 1c, there was a significant increase in secreted IL-10 protein in tumor cells co-cultured with B cells. These findings indicate that cross-talk between tumor cells and B cells promotes inflammatory signaling in tumor cells that may be associated with immunosuppression.

### TNBC cells enhance IgG4 expression by B lymphocytes

**Fig. 2:**
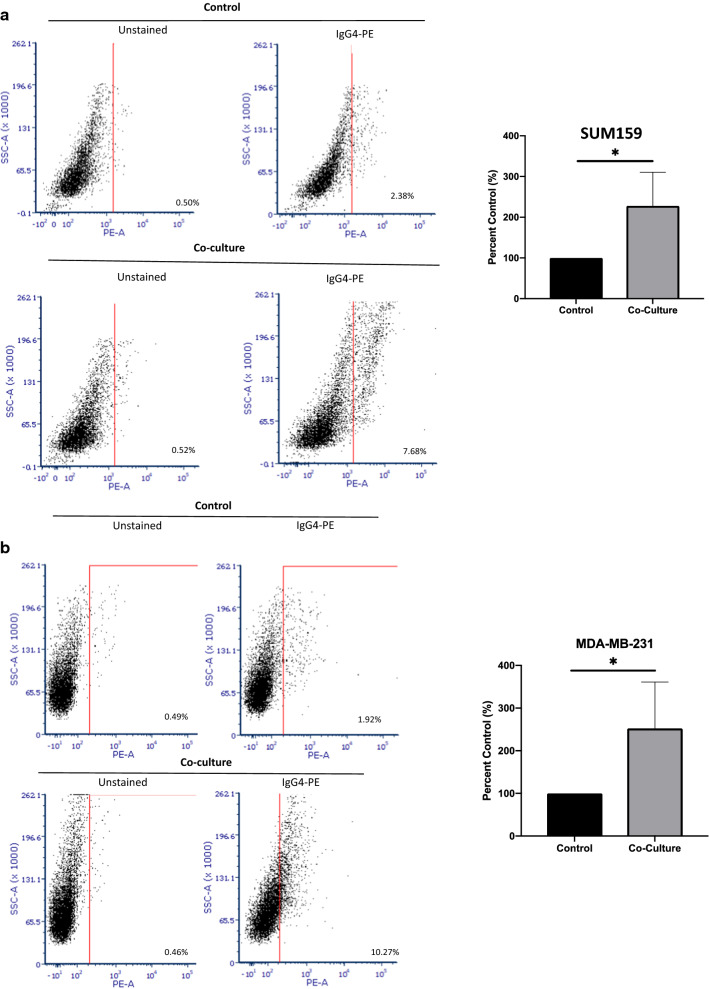

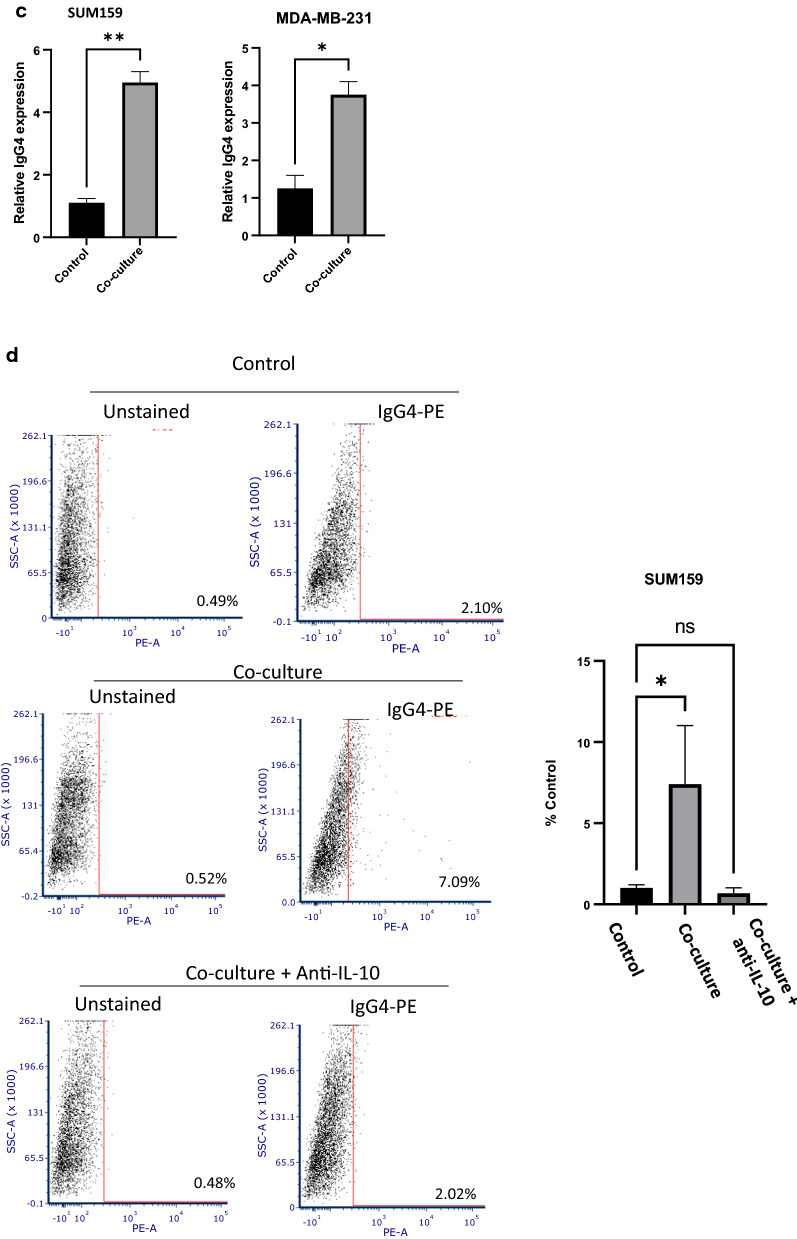

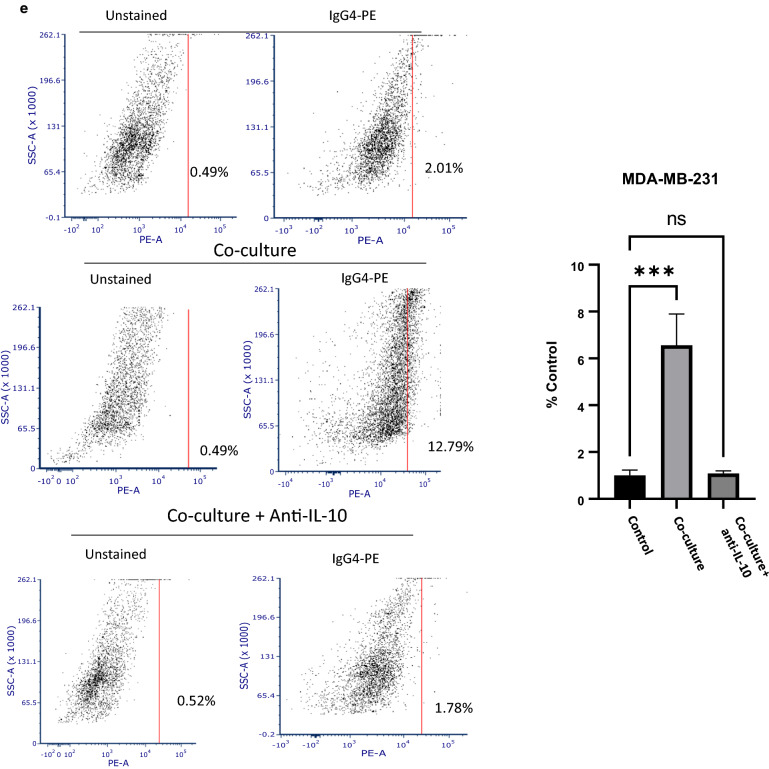
**a**, **b** Tumor cells induce IgG4 class switching in B cells. A representative flow cytometry experiment showing increased IgG4 + cells in **a** SUM159 and **b** MDA-MB-231 cells co-cultured with primary peripheral B cells. Activated primary B cells were cultured with or without tumor cells for 5–7 days and analyzed by flow cytometry. Graphs show results of three separate experiments. **c** Graph represents a relative increase in IgG4 protein expression as detected by ELISA in SUM159 and MDA-MB-231 cells co-cultured with B cells compared to control tumor cells. B cells were cultured with tumor cells for 5 days, and then transferred to a new flask. Cells were cultured for 2 weeks and supernatant was collected an analyzed by ELISA. This experiment was performed in duplicate. Error bars represent standard deviation of technical replicants. (**d**, **e**) A representative flow cytometry dot- plot showing increased IgG4 positive cells upon co-culture with an EBV-transformed B cell line and (**d**) SUM159 cells or (**e**) MDA-MB-231 cells. Activated B cells were co-cultured with or without tumor cells and a blocking IL-10 antibody for 5–7 days and analyzed by flow cytometry. Increase in IgG4 class switching is prevented by addition of an anti-IL-10 antibody during co-culture. Graphs show the mean of three independent experiments. Error bars represent standard deviations Significance is indicated by asterisk, ****p < 0.0001, ***p < 0.001, **p < 0.01, *p < 0.01, ns p > 0.05

IL-10 expression is often present at sites of chronic inflammation and promotes immunosuppression of humoral responses through induction of isotype switching to IgG4. Therefore, we sought to determine if TNBC could alter the isotype profile of B cells. We performed an in vitro assay to determine if IgG4 class switching is differentially induced upon activation of B cells co-cultured with TNBC cell lines compared to controls. Co -culture of both MDA-MB-231 cells and SUM159 with primary peripheral B cells isolated from TNBC patients resulted in significant increases of IgG4 + cells, suggesting increased IgG4 class switching in response to TNBC cell co-culture (Fig. 2a and b). To confirm IgG4 class switching, an IgG4 specific ELISA assay was performed in EBV-transformed B cells co-cultured with tumor cells. Significantly higher levels of IgG4 were observed in B cell supernatants two weeks after co-culture with TNBC cell lines (Fig. 2c). Likewise, EBV B cells were shown to undergo in vitro isotype switching similar to peripheral B cells upon co-culture with TNBC cell lines in flow cytometry experiments (Fig. 2d and e). To examine the role of IL-10 in class switching, we examined class switching in EBV-transformed B cells upon co-culture with TNBC with and without the addition of an IL-10 blocking antibody. As shown in Fig. 2d and e, the addition of the anti-IL10 antibody significantly blocked IgG4 class switching in co-cultured cells. These findings indicate that TNBC can induce IgG4 class switching in B cells in an IL-10 dependent manner.

### Presence of IgG4 + B cells, IL-10 and IL-4 in the tumor microenvironment of TNBC

**Fig. 3:**
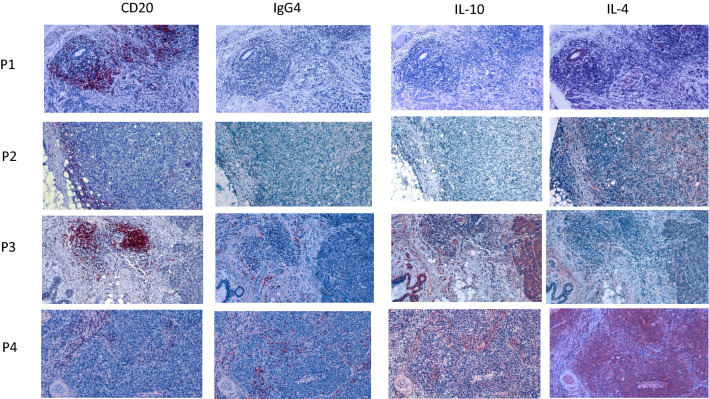
Correlation of tumor expressed inflammatory cytokines and IgG4 + B cells in TNBC tissues. Immunohistochemical analysis of serial tissue sections taken from 4 patients (p1-p4). Tissues were independently stained for CD20, IgG4, IL-10 and IL-4 (red stain). Nuclei were counterstained with hematoxylin (blue). Images were taken with a 10× objective

**Fig. 4:**
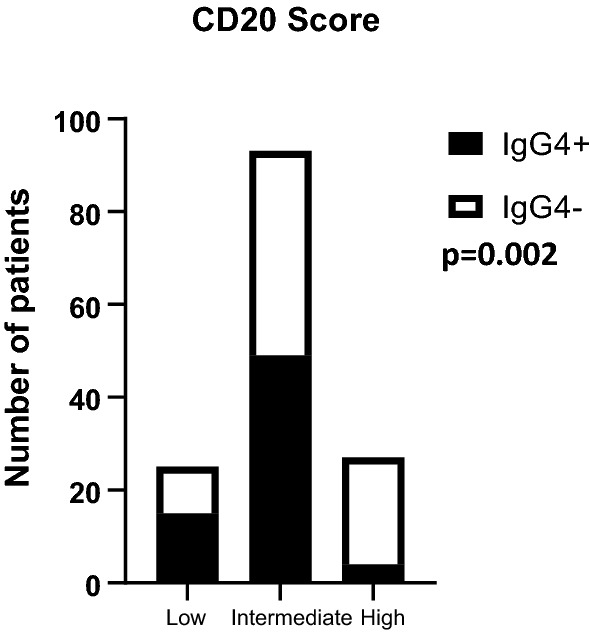
IgG4 + cells correlate with medium–low B cell density. Quantification of CD20 positive cells in tissue sections by low, medium and high density. IgG + cells are represented in black, IgG4- cells are represented in white

To investigate the presence of IgG4 and inflammatory cytokines in TNBC, we sought to evaluate protein expression by immunostaining tumor specimens from 152 triple negative tumors. Table 2 provides a summary of clinical data and patient characteristics. In order to quantify IgG4 + B cell and total B cell infiltration in TNBC tissues, serial sections of tissue microarrays were stained by immunohistochemistry for the pan-B cell marker CD20, IgG4, IL-10 and IL-4 (Fig. 3). The majority of tumors contained CD20 + B cells. Only 5 section (3.3%) scored negative for CD20 + B cells. The average density of B cells was 30.1%. A low density of B cells was defined to be those tumors less than one standard deviation from the mean, while high density was defined as a B cell density more than one standard deviation above the mean. Twenty tumors (13.3%) had low density, 93 (62%) had intermediate density and 27 (18%) had high density. IgG4 + cells were more often found in tumors with low or intermediate B cells (Fig. 4, p = 0.002). Tumor cell IL-10 staining was detected in 68 (45%) patients with high expression observed in 42 patients. Tumor cell IL-4 staining was detected in 102 tumors (68%), with high expression observed in 75 patients.

**Table 2:** Patient characteristics

	Number	Percent
Race		
White	82	53.95
Black	48	31.58
Other	1	0.06
Unknown	21	13.81
Grade		
1	2	1.98
2	26	17.11
3	116	76.31
Unknown	8	5.26
Stage		
1	42	27.63
2A	17	11.18
2b	31	20.39
3A	21	13.8
3B	15	0.98
3C	9	5.92
4	2	1.31
Unknown	15	9.86

Age	Mean	Range
	62	32–93

### IgG4 + B cells correlate with IL-10 expression, higher stage and poor outcomes

Prior studies indicate that IL-4 and IL-10 polarize IgG4 class switching [30], and that IgG4 + B cells are increased in advanced cancer [28], and associate with poor patient survival [29, 30] and increased recurrence [34]. Therefore, we compared immunohistochemically stained markers and clinicopathological characteristics using Spearman’s correlation analysis (Table 3). We observed no correlation of CD20 + B cell infiltration and stage or grade. Interestingly, a weak negative correlation was found between IgG4 and CD20 + B cells density (r = − 0.190, p = 0.020) as well as stage and B cell density (− 0.199, p = 0.021) confirming previous findings that a high B cell density is associated with better prognosis [38]. A correlation was also observed between IgG4 staining and tumor expressed IL-10 (r = 0.0.488, p = 0.000), and IL-4 (r = 0.262, p = 0.001), as well as between IL-10 and tumor stage (IL-10 r = 0.254, p = 0.016). Interestingly, IL-10 expression was significantly higher in blacks than non-hispanic whites (r =  − 0.176; p = 0.031).

**Table 3:** Correlation matrix

	Age	Race	Grade	Stage	CD20 Score	IgG4 score	IL-10 score	IL-4 score
Age								
Spearman’s r	1	− 0.295**	0.048	− 0.001	− 0.169	− 0.086	0.033	− 0.027
Sig. (2-tailed)	**0.001**	0.588	0.995	0.055	0.328	0.712	0.766	
Race								
Spearman’s r	− 0.295**	1	0.109	− 0.234**	0.015	− 0.058	− 0.176*	0.070
Sig. (2-tailed)	**0.001**	0.184	**0.007**	0.857	0.481	**0.031**	0.401	
Grade								
Spearman’s r	0.048	0.109	1	0.155	0.031	0.081	0.113	0.057
Sig. (2-tailed)	0.588	0.184	0.075	0.705	0.327	0.171	0.496	
Stage								
Spearman’s r	− 0.001	− 0.234**	0.155	1	− 0.199*	0.050	0.254*	0.106
Sig. (2-tailed)	0.995	**0.007**	0.075	**0.020**	0.567	**0.016**	0.233	
CD20 Score								
Spearman’s r	− 0.169	0.015	0.031	− 0.199*	1	− 0.190*	− 0.202*	− 0.046
Sig. (2-tailed)	0.055	0.857	0.705	**0.020**	**0.020**	**0.013**	0.579	
IgG4 Score								
Spearman’s r	− 0.086	− 0.058	0.081	0.050	− 0.190*	1	0.448**	0.262**
Sig. (2-tailed)	0.328	0.481	0.327	0.567	**0.021**	**0.000**	**0.001**	
IL-10 Score								
Spearman’s r	0.033	− **0.176**	0.113	0.254*	0.202*	0.448**	1	0.310
Sig. (2-tailed)	0.712	**0.031**	0.171	**0.016**	**0.013**	**0.000**	0.714	
IL-4 Score								
Spearman’s r	− 0.027	0.070	0.057	0.106	− 0.046	0.262**	0.310	1
Sig. (2-tailed)	0.766	0.401	0.496	0.233	0.579	**0.001**	0.714	

**Correlation is significant at the 0.01 level (2-tailed)

*Correlation is significant at the 0.05 level (2-tailed)

**Fig. 5:**
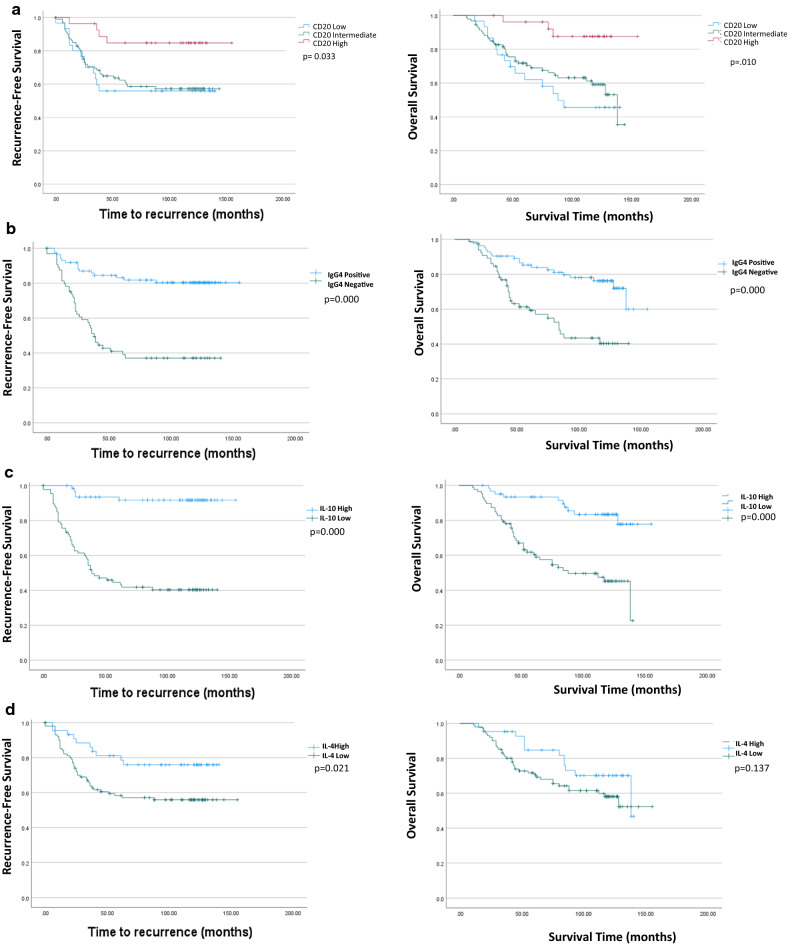
Survival analysis of TNBC patients by expression of IgG4 + B cells, and inflammatory cytokines. Kaplan Meyer survival curves showing recurrence free and overall survival outcomes for patients with (**a**) high, intermediate or low densities of CD20, (**b**) IgG4 + or IgG4- B cells (**c**) high or low IL-10 expression (**d**) High or low IL-4 expression

To assess the effects of IgG4 + B cells on patient outcomes, Kaplan-Meyer analysis was performed. Consistent with previous studies, high B cell density was found to have significantly better RFS (p = 0.033) and OS (p = 0.010) than intermediate or low B cell density (Fig. 5a). Patients with high IgG4 + cells had significantly worse RFS (p = 0.000) and OS (p = 0.000) (Fig. 5b). Furthermore, high tumor expression of IL-10 and IL-4 resulted in lower recurrence free survival (p = 0.000 for IL-10; p = 0.021 for IL-4) (Fig. 5c and d). While high IL-10 expression resulted in worse OS (= 0.000), high expression of IL-4 had no significant effect on OS (p = 0.137). In a multi-variant analysis, IgG4 positivity (p = 0.001; HR = 3.366; 95% CI 1.607–7.050), tumor IL-10 (p = 0.000; HR = 7.195; 95% CI 2.418–21.411) and tumor stage and tumor stage (p = 0.000; HR = 1.442; 95% CI 1.0247–1.667) were independently associated with a shorter RFS. However, only tumor stage (p = 0.000; HR = 1.329; 95% CI 1.151–1.534) and IL-10 (p = 0.005; HR = 4.1401; 95% CI 1.538–10.934) were independently associated with a shorter OS (Table 4).

**Table 4:** Multivariate analysis

	Sig	HR	95% CI for HR
Recurrence free survival			
Race	0.791	1.095	0.560–2.140
Grade	0.34	1.128	0.881–1.445
Stage	**0.000**	1.442	1.247–1.667
CD20 score	0.231	0.72	0.420–1.236
IgG4	**0.001**	3.336	1.607–7.0560
IL-10	**0.000**	7.195	2.418–21.411
IL-4	0.279	1.535	0.707–3.337
Overall survival			
Race	0.604	0.83	0.411–1.678
Grade	0.931	1.014	0.736–1.398
Stage	**0.000**	1.329	1.151–1.534
CD20 score	0.211	0.706	0.409–1.218
IgG4	0.074	2.541	1.204–5.336
IL-10	**0.005**	4.101	1.538–10.934
IL-4	0.528	1.283	0.592–2.779

## Discussion

In this study, we report that presence of IgG4 + B cells in the tumor microenvironment of TNBC associates with increased tumor recurrence and poor patient survival. We also report that presence of IgG4 + B cells correlates with tumor expression of IL-10 and trends with expression of IL-4 by tumors. As IL-10 and IL-4 are known to promote class switching to IgG4 [22, 39], these findings indicate that TNBC may create a tumor microenvironment that supports class switching to the IgG4 subtype.

TIB have been shown to undergo class switching and somatic hypermutation at a higher frequency in TNBC than other breast cancer subtypes [40, 41]. Although an increase in IgG1 has been associated with better prognosis [41], the role of IgG4 has is unclear in breast cancer. However, the association between IgG4 and poor outcomes has been demonstrated in melanoma and other tumor types [24, 28, 29]. Consistent with our findings, these studies have also shown TIB expressing IgG4 correlate with the presence of Th2 cytokines [29].

Based on our studies, it is yet to be determined if IgG4 in TNBC actively contributes to poor patient outcomes, or if it is a bystander in a larger process. There is evidence in melanoma that IgG4 hinders tumor associated antigen targeted IgG1, regardless of if the IgG4 antibody was antigen specific or not, indicating that IgG4 may actively impede an anti-tumor antibody response regardless of antigen specificity [31]. In addition to promoting IgG4 class switching, IL-10 is an immunosuppressive cytokine, and can inhibit the ability of dendritic cells and macrophages to activate CD4 + helper T cells [42]. In breast cancer, IL-10 expression in tumors positively correlates with locally advanced disease, higher tumor grade, and hormone-receptor negativity [43, 44]. This finding is different in non-TNBC, where IL-10 is found to be a good prognostic indicator of disease-free survival (DFS) [45]. In melanoma, IL-10 expression by tumor cells associates with melanoma progression [46], and high serum IL-10 associates significantly with worse OS [47]. Therefore, it is possible that IL-10 expression by tumor cells may also be a driver of poor outcomes in TNBC, and this may be independent of IgG4 + B cells. This is supported by our multi-variant analysis where only stage and tumor IL-10 were predictive of shorter OS. Although not examined in the scope of this study, significant expression of IL-10 was also observed in TIB and may play a role in directing anti-tumor immune responses as well. Further study is needed to determine the role of B cell expressed IL-10 in TNBC and if IgG4 + B cells serve as regulatory B cells.

In this study, we find that B cells enhance inflammatory responses, including IL-10 expression in TNBC cells. Cross-talk between tumor cells and TIB may help to shift the immune response towards and immunosuppressive state through induction of IL-10 expression and release by tumor cells. Enhanced IL-10 expression has also been reported in melanoma cells co-cultured with B cells [30]. The mechanism driving IL-10 gene expression in tumor cells is not yet fully understood. Gene expression studies show that the transcription factors AP-1 and NFκB are important in driving transcription of IL-10, but the majority of studies have been conducted in immune cells [48]. Further study is needed to determine if IL-10 expression by TNBC is dependent upon B cell induced NFκB or other inflammatory signaling mechanisms. Understanding what drives IL-10 expression in TNBC may reveal potential therapeutic targets for TNBC.

The ability of tumor cells to direct TIB behavior has been previously described in melanoma and lung cancer [49, 50]. Co-culture with supernatants of melanoma cells was able to induce activation of NFκB and expression of inflammatory cytokines in B cells as well as isotype switching to IgG4. Likewise, co-culture of LPS-stimulated lung tumor cells was shown to induce IL-10 expression and a regulatory phenotype in peripheral B cells. However, these studies did not investigate effects on tumor cells. Our studies show that that co-culture also results in increased expression of inflammatory cytokines by tumor cells. Our findings indicate that significant cross-talk may occur between B cells and tumor cells that may help to shape immune responses. Whether or not enhanced inflammatory signaling in TNBC occurs as a direct consequence of B cell expressed inflammatory cytokine expression remains to be determined.

The role of humoral immune responses in breast cancer is unclear. Recently, high densities of TIB were found to associate with good prognosis in breast cancer [38]. Garaud et al., reported TIB cells with a germinal center phenotype (CD19^+^CD38^high^IgD^−^) were more often found in breast cancers with high TIB density and these cells were found to associate with tertiary lymphoid structures and maintain functionality. Analysis of secreted immunoglobulins in the supernatants of TIB showed increases in IgG1, IgG2 and IgG3 with increasing TIB density, with a higher concentration of IgA obtained from tissues with intermediate B cell infiltration [38]. Likewise, increased clonal expansion of IgG + cells has been reported in stromal clusters [35]. These findings indicate that TIB isotype may be associated with B cell density or other features of the tumor microenvironment. In our study, IgG4 expression was associated with low to medium density of TIB. Of note, IgG4 expressing B cells were more often located intratumorally and diffusely spread throughout the tumor microenvironment, as opposed to being located in B cell aggregates or at the invasive margin. This spatial distribution may promote more interactions with tumor cells as opposed to other immune cells as has been previously described [51]. The association to tertiary lymphoid structures was not examined in our study and it is unknown if these structures are needed for isotype switching to IgG4 or if this polarization occurs elsewhere. Furthermore, the exact phenotype and clonality of IgG4^+^ B cells remains to be characterized.

## Conclusions

Our results support that IgG4 expression by B cells is induced through signaling from TNBC cells, and presence of IgG4 + B cells in the TNBC microenvironment associate with worse patient OS, BCSS, and RFS. We show that infiltration of IgG4 + B cells correlate with intermediate but not high densities of B cell infiltration, IL-10 expression by tumor cells, and higher stage. Understanding the contribution of IgG4 + cells to the immune microenvironment of TNBC may reveal ways in which TIB can contribute to tumor growth and provide new targets to increase the immune response to TNBC. As different diagnostic criteria may exist between institutions for TNBC, and there are various subclasses within TNBC, these findings should be confirmed in a larger study population and in the context of heterogeneous subclasses of TNBC.

## Supplementary Information

**Additional file 1: Figure S1.** Immunohistochemical analysis of TNBC tissue sections using an isotype matched primary control antibody. Slides were stained with a mouse (top) or rabbit (bottom) IgG control antibody following the same protocol used for detection of cytokines, except the primary antibody was an isotype control antibody. Nuclei are stained with hematoxylin.

## Data Availability

The datasets used and/or analyzed during the current study are available from the corresponding author on reasonable request.
