# Supplementary figures and images for: Tumor-B-cell interactions promote isotype switching to an immunosuppressive IgG4 antibody response through upregulation of IL-10 in triple negative breast cancers
Source: J Transl Med. 2022 Mar 7;20:112. doi: 10.1186/s12967-022-03319-5 (PMC8900352; doi:10.1186/s12967-022-03319-5)

## Slide 1
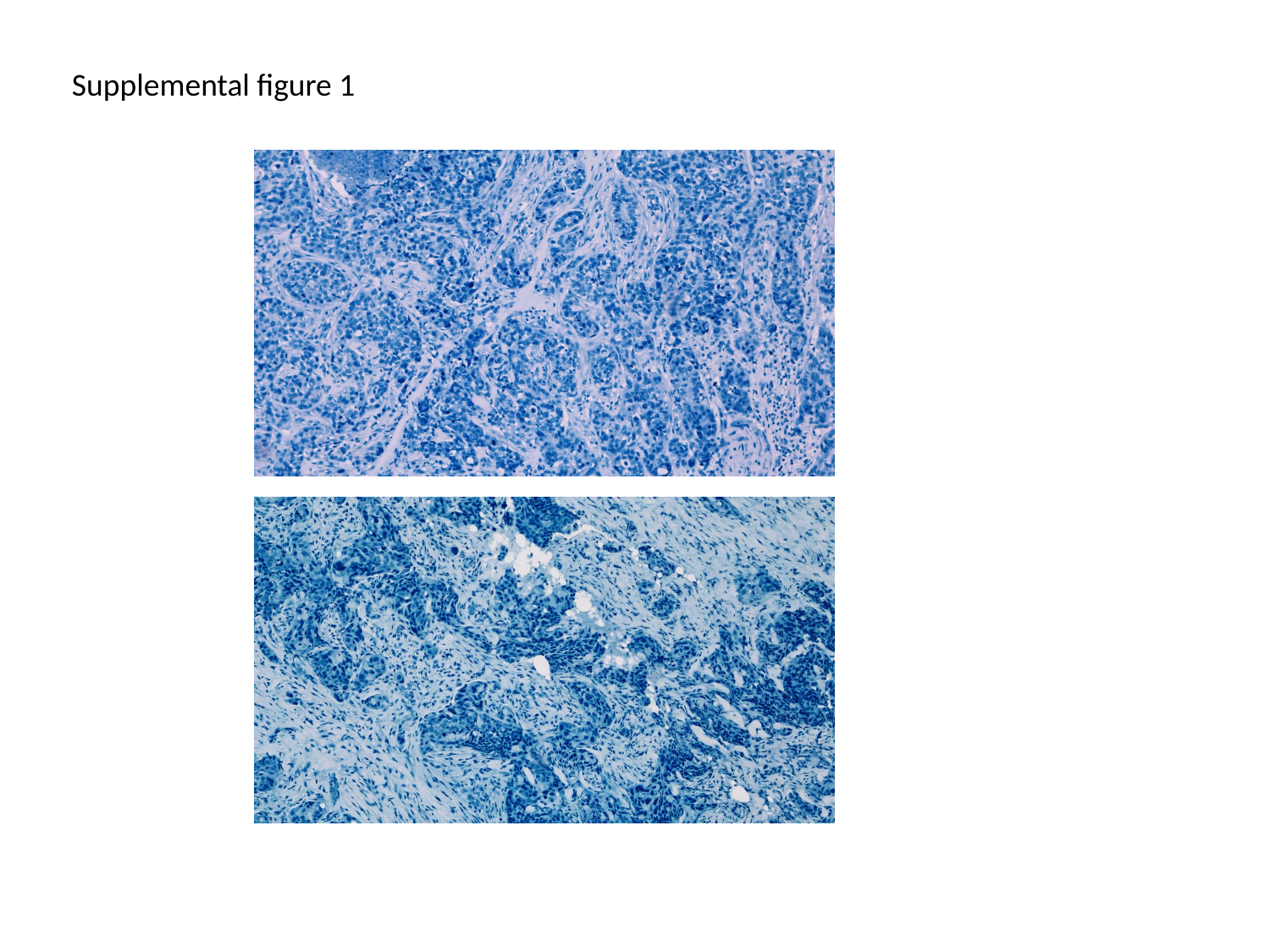

Supplemental figure 1

Supplement: Supplementary file 1 — Additional file 1: Figure S1. Immunohistochemical analysis of TNBC tissue sections using an isotype matched primary control antibody. Slides were stained with a mouse (top) or rabbit (bottom) IgG control antibody following the same protocol used for detection of cytokines, except the primary antibody was an isotype control antibody. Nuclei are stained with hematoxylin. [file 12967_2022_3319_MOESM1_ESM.pptx]
